# Venetoclax in association with decitabine as effective bridge to transplant in a case of relapsed early T‐cell lymphoblastic leukemia

**DOI:** 10.1002/ccr3.3041

**Published:** 2020-07-07

**Authors:** Elisabetta Zappone, Emanuele Cencini, Marzia Defina, Anna Sicuranza, Alessandro Gozzetti, Sara Ciofini, Donatella Raspadori, Bianca Mecacci, Monica Bocchia

**Affiliations:** ^1^ Hematology Unit Azienda Ospedaliera Universitaria Senese University of Siena Siena Italy

**Keywords:** acute lymphoblastic leukemia, allogeneic transplantation, decitabine, relapse, venetoclax

## Abstract

A case of an early‐relapsed high‐risk T‐ALL with high BCL‐2 expression on leukemic blasts was successfully treated with decitabine and venetoclax, achieving a CR. We suggest decitabine and venetoclax should be synergistic in BCL2‐positive ALL.

## Graphical abstract

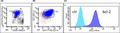

## 1 INTRODUCTION

We found a high BCL‐2 expression on leukemic blasts of an early‐relapsed high‐risk T‐ALL, and thus the patient started decitabine and venetoclax, achieving a CR. The patient underwent allo‐SCT; CR was maintained at last follow‐up. We postulated a synergistic action of venetoclax‐decitabine, enhanced by high BCL‐2 expression on leukemic blasts.

T‐cell acute lymphoblastic leukemia (T‐ALL) is an aggressive hematological disease often characterized by a poor prognosis despite high‐dose chemotherapy.^1^ Early T‐ALL with negative CD1a antigen is considered high‐risk disease, especially in adult patients, in which long‐term disease control is achieved in a limited proportion of cases even when they are treated with pediatric‐like dose‐dense chemotherapy programs.^2^ They have a strong indication for allogeneic stem cell transplantation (allo‐SCT), that represents the only strategy to obtain long‐term remission and survival.^3^

Moreover, therapeutic options for relapsed patients demonstrate a limited possibility to achieve a complete remission (CR) and are often burdened by important treatment‐related toxicity, such as grade 3‐4 infections, finally resulting in fewer possibilities to proceed to allo‐SCT.^2^

There is a growing evidence that the inhibition of B‐cell lymphoma 2 (BCL‐2), an antiapoptotic protein, could have therapeutic effects in many hematological malignancies, both in preclinical studies and clinical protocols.^4^ The oral highly specific BCL‐2 inhibitor venetoclax is approved for the treatment of chronic lymphocytic leukemia (CLL); it has also recently received FDA approval, in combination with azacytidine, decitabine, or low‐dose cytarabine, for the treatment of newly diagnosed acute myeloid leukemia (AML) patients who are age 75 years or older, or who have comorbidities that preclude to receive intensive chemotherapy, based on phase I‐II studies in which this combination was very effective and well tolerated.^5^, ^6^

High BCL‐2 expression and high sensitivity to venetoclax‐mediated BCL‐2 inhibition was reported in a preclinical study performed on T‐ALL cell lines.^7^ Clinical response to venetoclax was recently reported in 2 early T‐cell precursor relapsed/refractory ALL patients.^8^ Furthermore, hypomethylating agents demonstrated a potential role in ALL treatment, suggesting a rationale for combination strategies.^9^ According to this background, venetoclax in combination with decitabine was successfully used to treat a patient who experienced T‐ALL relapse after allo‐SCT.^10^

## 2 CASE REPORT

Here, we report the case of an adult patient who experienced an early relapse of high‐risk T‐ALL just before a planned allo‐SCT, who was successfully treated with decitabine in combination with venetoclax.

A 56‐year‐old woman was admitted to emergency department with significant dyspnea. The patient's medical history included close follow‐up for bilateral breast fibroadenoma since she was 20 years old. CT scan showed the presence of a bulky mediastinal mass with multiple enlarged cervical lymph nodes and massive pleural effusion. Blood cell counts documented leukocytosis (WBC 18.7 × 10^9^/L) with 90% ungranulated blasts, mild anemia (Hb 10.2 g/dL), and thrombocytopenia (platelet 91 × 10^9^/L). Bone marrow (BM) examination showed a hypercellular marrow with >90% of lymphoid blasts (morphologically defined as FAB L2) that expressed terminal deoxynucleotidyl transferase (TdT), cyCD3, CD34, CD71 and lacked expression of CD1a and myeloperoxidase (MPO); fluorescence in situ hybridization (FISH) and karyotype were normal, molecular analysis was negative for FLT3 mutations. Cytologic examination of pleural effusion revealed lymphoblast cells positive for CD117, CD34, and TdT. According to WHO classification, a diagnosis of early T‐ALL was made.

The patient started a pediatric‐like regimen, as described in the NILG/GIMEMA protocol, with peg‐asparaginase instead of the native compound.^11^ A morphological CR was achieved after 1st cycle, minimal residual disease (MRD) assessment by flow cytometry (0.0026%) confirmed the optimal response; thus, a donor search was immediately activated. The patient proceeded to consolidation with the 2nd and 3rd cycle of the planned treatment, while a 10/10 human leukocyte antigen compatible matched unrelated donor was found and an allo‐SCT was scheduled.

PET scan performed after the 3rd cycle (as part of response assessment before transplant) revealed a right breast nodule. Histology report showed an infiltrating ductal carcinoma. After quadrantectomy and sentinel lymph node biopsy a final diagnosis of pT1bN1, G3, estrogen receptor positive, human epidermal receptor (Her)‐2 positive, progesterone receptor negative breast cancer was made.

After multidisciplinary discussion of the case, we agreed to continue the ALL treatment with intercalating administration of anti‐Her‐2 monoclonal antibody trastuzumab. The patient completed cycles 4‐8 of the NILG/GIMEMA regimen and received six trastuzumab infusions, maintaining a CR for ALL with MRD <0.002%; breast cancer treatment was successfully completed with local radiotherapy (RT).

**FIGURE 1:**
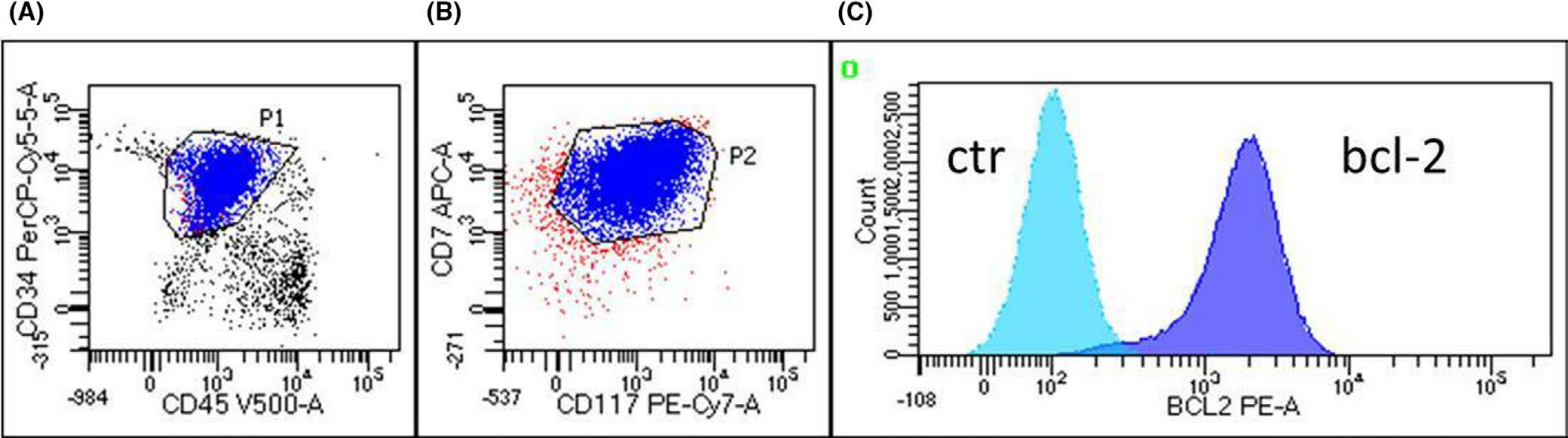
Acute lymphoblastic leukemia population and expression of bcl‐2 protein. Identification of ALL population using antigen expression is represented in panel A and panel B; bcl‐2 and isotypic control histograms are illustrated in panel C. High bcl‐2 expression in panel c histogram is shown in blue

In view of patient's very good performance status and the high risk of her leukemia, allo‐SCT program was confirmed; unfortunately, an abrupt hematological relapse occurred 2 weeks after completing breast RT, with 80% of bone marrow lymphoblast infiltration. Initial early T‐ALL phenotype was confirmed by flow cytometry. According to promising preclinical data and pivotal case reports about venetoclax efficacy in T‐ALL,^7^, ^8^, ^10^ we decided to test BCL‐2 expression on the leukemic blasts, and we found a high expression (Figure 1). This finding encouraged us to start salvage treatment with standard‐dose decitabine, 20 mg/m^2^ for 5 days every 28 days in combination with venetoclax, daily dose 400mg, after a brief rump up of 6 days (initial daily dose 100 mg). The patient signed informed consent, and the off‐label drug use received approval from the Hospital Ethical Committee.

No tumor lysis syndrome was observed, a dose reduction to daily 200 mg was necessary when concomitant antifungal prophylaxis with posaconazole was indicated because of grade 4 neutropenia. At day 28, BM aspirate showed significant blast count reduction (15% compared to 80% on the pretreatment evaluation). The 2nd cycle was complicated by febrile neutropenia, and venetoclax was shortly interrupted until neutrophil recovery. BM aspirate at day 28 demonstrated morphologic CR, and MRD by immunophenotyping was 0.02%; CT scan showed no signs of lymph nodes or breast cancer recurrence. The patient continued venetoclax alone until 1 week before allo‐SCT, performed 6 weeks later while maintaining CR. Conditioning regimen included cyclophosphamide, fludarabine, and thiotepa with cyclosporine, methotrexate, and antithymocyte globulin, as graft‐vs‐host disease prophylaxis. No significant treatment‐related complications have occurred so far, and the patient maintains CR 3‐month post‐transplant follow‐up.

## 3 DISCUSSION

Our case suggests venetoclax in association with decitabine could represent a feasible treatment option for relapsed/refractory T‐ALL with good efficacy and manageable toxicity.

Despite the role demonstrated in lymphoproliferative disease such as CLL and MCL, the employment of venetoclax in ALL is reported in only three published cases.^8^, ^10^ Maturation stage of T‐ALL cells determines BCL‐2 vs BCL‐XL dependence and sensitivity to BCL‐2 inhibition.^12^ Venetoclax use is supported by in vitro studies on T‐ALL‐derived cell lines and ex vivo patients derived blasts, suggesting a rationale for a novel therapeutic strategy.^7^

Our case is of particular interest because decitabine in combination with venetoclax led to a rapid (after 2 cycles) and profound CR, allowing the patient to undergo allo‐SCT. As hypomethylating agents alone were shown scarcely effective in T‐ALL,^13^ we postulated a synergistic action of venetoclax‐decitabine combination,^14^ that could be enhanced by the high BCL‐2 expression on T‐cell leukemic blasts shown in this patient.

In conclusion, high‐risk‐relapsed/refractory early T‐ALL is a great challenge for the hematologist because of the possibilities to achieve and maintain a CR are limited. Since early T‐cell precursors may express high levels of BCL‐2, like in our patient, the use of venetoclax is intriguing. The possible synergistic effect with hypomethylating agents, previously hypothesized in AML, could be effective in ALL cases; it could pave the way toward a new therapeutic option to investigate in future clinical trials.

## CONFLICTS OF INTEREST

The authors declare no conflicts of interest.

## AUTHOR CONTRIBUTIONS

EZ and EC: conceived and wrote the manuscript. MD, SC, AG, and BM: collected and analyzed clinical data. DR and AS: performed and analyzed biological data. MB: conceived, revised, and finally approved the manuscript. All authors revised and approved the final manuscript.
